# Tea, Coffee, and Liquor

**Published:** 1870-04

**Authors:** 


### TEA, COFFEE, AND LIQUOR.

The world will have stimulants until mankind shall be elevated to the sublime principles of the Apostle Paul, who declared that although he had a right to eat meat of a certain kind, yet for the sake of not disturbing the consciences of the weak-minded, who thought that such an indulgence was sinful, he would not partake of it "while the world standeth."

If stimulants will be used or must be used, it is important to educate the public mind to the selection of those which are least hurtful; of this class are tea and coffee. Of the two, tea is the more universally applicable, and the least hurtful, although their distinctive elements are identical in their chemical constituents, according to the light of the most advanced chemistry.

Multitudes have found that coffee makes them bilious; it muddles the brain, causes heaviness, dullness, headache, nausea, and other discomforts, — not to all certainly, for multitudes have used coffee all their days without being able to perceive any appreciable ill result.

Of all the forms and designations of tea brought from China, the "English Breakfast Tea" is the least objectionable, the least apt to disturb the nervous system.

The great error in the use of stimulants is,

First, in increasing the strength,
Second, increasing the frequency,
Third, increasing the quantity.

But so few persons have force of character enough to keep within these bounds, that the aggregate of the world's happiness would be immeasurably increased, and its miseries and crimes diminished to such an amazing extent, by total abstinence, that it would seem as if a sense of humanity alone ought to be sufficient to impel every generous and noble nature to rise to St. Paul's moral heroism, and say, For the sake of humanity, for the sake of my soul, I will not use a drop of stimulus as long as the world standeth.

Thus far as to those who are well. There are various conditions in human life which require stimulants in various forms; in many debilitated conditions of the system they are of advantage.

If a man, sick or well, comes home to his din ner cold, and hungry, and wearied, and worried, a cup of hot tea, drank during the fore part of the meal, is of great physiological benefit, for it warms up the system by the heat of the beverage being conveyed direct to the centre of life; it rouses the stomach to healthful action; it arrests the depressing and failing powers of life, energizes the circulation, and wakes up the activities of the brain and moral sentiments. The Brompton Hospital, of London, was originally projected for the special treatment of consumption and kindred diseases, and from the observations there made in the course of many years, it has been decided by the officials in charge that in these diseases tea and coffee exercise a far more beneficial and salutary effect on the system than any form of intoxicating drinks.

